# Surface Micro Discharge–Cold Atmospheric Pressure Plasma Processing of Common House Cricket *Acheta domesticus* Powder: Antimicrobial Potential and Lipid-Quality Preservation

**DOI:** 10.3389/fbioe.2021.644177

**Published:** 2021-07-02

**Authors:** Maria C. Pina-Pérez, Dolores Rodrigo, Christoph Ellert, Michael Beyrer

**Affiliations:** ^1^Departamento de Microbiologia y Ecología, Universitat de València, Valencia, Spain; ^2^School of Engineering, Institute of Life Technologies, University of Applied Sciences and Arts Western Switzerland (HES-SO VS), Sion, Switzerland; ^3^Departamento de Conservación y Calidad, Instituto de Agroquimica y Tecnología de Alimentos (IATA-CSIC), Valencia, Spain

**Keywords:** insect powder, *Acheta domesticus*, cold plasma, microbial decontamination, lipids, food safety, non-thermal processing

## Abstract

The growing world population and the need to reduce the environmental impact of food production drive the exploration of novel protein sources. Insects are being cultivated, harvested, and processed to be applied in animal and human nutrition. The inherent microbial contamination of insect matrices requires risk management and decontamination strategies. Thermal sterilization results in unfavorable cooking effects and oxidation of fatty acids. The present study demonstrates the risk management in *Acheta domesticus* (home cricket) powder with a low-energy (8.7–22.0 mW/cm^2^, 5 min) semi-direct surface micro discharge (SMD)–cold atmospheric pressure plasma (CAPP). At a plasma power density lower than 22 mW/cm^2^, no degradation of triglycerides (TG) or increased free fatty acids (FFA) content was detected. For mesophilic bacteria, 1.6 ± 0.1 log_10_ reductions were achieved, and for *Enterobacteriaceae*, there were close to 1.9 ± 0.2 log_10_ reductions in a layer of powder. Colonies of *Bacillus cereus, Bacillus subtilis*, and *Bacillus megaterium* were identified *via* the mass spectral fingerprint analyzed with matrix-assisted laser desorption/ionization time of flight (MALDI-TOF) mass spectrometry (MS). The spores of these *Bacillus* strains resisted to a plasma power density of 22 mW/cm^2^. Additional inactivation effects at non-thermal, practically non-oxidative conditions are supposed for low-intensity plasma treatments combined with the powder’s fluidization.

## Introduction

Insects are recognized as a valuable source of proteins, poly-unsaturated fatty acids (PUFA), vitamins, minerals, and nutritional fibers (van Huis et al., 2013). The house cricket (*Acheta domesticus* L.) belongs to the protein-rich insects of specific interest (see Table 1). Although edible insects have been consumed from ancient times by several civilizations worldwide, the consumption in the Western countries was emerging just close to 2010 (van Huis et al., 2013; Mason et al., 2018). Since 2013, insects were used more frequently in whole or parts of the insects, in formulations for humans and animals (Borrelli et al., 2017). In 2018, the European Union recognized insects as novel foods (EU Novel Foods regulation, 2015/2283). However, eating whole insects is still unfamiliar to most people in Western countries. So, most food companies offer flours or powders of extracted proteins to develop novel products (Megido et al., 2016; Dobermann et al., 2017).

**TABLE 1:** Composition of insects used as a protein source in the human feed (based on Williams et al., 2016).

**Insect**	**Common name**	**Crude protein (%)**	**Crude fat (%)**	**Fiber (%)**
*Bombyx mori*	Silkworm	64.7	20.8	
*Heliothis zea*	Corn earworm	18.2		
*Spodoptera frugiperda*	Fall armyworm	59.3	11.7	12.4
*Galleria mellonella* (larva)	Waxworm	34.0	60.0	8.1
*Xyleutes redtenbacheri* (larva)	Carpenter moths	48		6.0
*Oileus rimator* (larva)	Beetle	36.0		15.0
*Rhyncophorus ferrugineus* (larva)	Red palm weevil	20.7	44.4	
*Zophobas morio* (larva)	Darkling beetle	46.8	42.0	6.3
*Acheta domesticus* (adult)	House cricket	66.6	22.1	10.2
*Acheta domesticus* (nymph)		67.2	14.4	9.6
*Sphenarium histro* (nymphs and adults)	Grasshoppers	4.0		12.0
*Macrotermes bellicosus* (alate)	African termites	34.8	46.1	
*Apis mellifera* (larva)	European honeybee	40.5	20.3	1.3
*Drosophila melanogaster* (adult)	Common fruit fly	56.3	17.9	
*Hermetia illucens* (larva)	Black soldier fly	47.0	32.6	6.7

According to EFSA risk assessment published in October 2015 (EFSA, 2015), some microbiological and chemical hazards have been identified to be associated with ingredients derived from insects, depending on different factors: (i) production methods; (ii) what the insects are fed on; (iii) insect species; (iv) life cycle stage at which insects are harvested and consumed; and (v) the methods applied for processing. Specifically, using *A. domesticus* as an edible insect ingredient, the scientific concern is about both: (1) high total aerobic bacterial counts in crude materials and (2) survival of spore−forming bacteria following thermal processing (Fernández-Cassi et al., 2018).

Wet and dry thermal treatment was applied to decontaminate insect powders (Rumpold et al., 2014; Fasolato et al., 2018; Kröncke et al., 2018). Despite a possible protein denaturation and reduced applicability as a functional food ingredient, the thermal treatment results in considerable fatty acid oxidation (Tiencheu et al., 2013; Jeon et al., 2016). Rumpold et al. (2014) compared the decontamination of whole mealworm larvae with thermal and cold plasma processing. A remote plasma powered with a 1.2-kW microwave source resulted in 5 log_10_ reductions within 10 min.

Cold plasma was also proven as an effective method to inactivate spores from pathogenic bacteria on powdered food (Bußler et al., 2016; Beyrer et al., 2020a,b; Pina-Pérez et al., 2020). The inactivation of *Bacillus* spores embedded in a powder required a plasma power density of 10 mW/cm^2^ only when generating the plasma with a surface micro discharge (SMD) device (Beyrer et al., 2020a; Pina-Pérez et al., 2020). It was suggested that microorganisms be inactivated by etching through reactive oxygen and nitrogen species, altering of the cytoplasmic membrane, metabolic proteins, DNA, or photo-oxidation (Waskow et al., 2018; Pina-Pérez et al., 2020). However, plasma does not act selectively on bacterial cells, and active plasma species can trigger chemical reactions with food matrix components, mainly carbohydrates, proteins, and lipids (Chen et al., 2012; Meinlschmidt et al., 2016; Gavahian et al., 2018; Pan et al., 2019; Sharma and Singh, 2020).

The present study aims to evaluate SMD-CAPP technology potential to be used in *A. domesticus* crude powder’s decontamination. Due to the nutritional value of *A. domesticus* as a source of poly-unsaturated fatty acids (PUFA), the free fatty acid concentration was measured as an indicator of possible hydrolysis of TGs under CAPP processing suited for the inactivation of reasonable amounts of microorganisms.

## Materials and Methods

### Acheta Domestica Powder

Crude powder from *A. domesticus* was provided by Thailand Unique (Available at https://www.thailandunique.com/) (humidity 12% w/w).

### Surface Micro Discharge–Cold Atmospheric Pressure Plasma (SMD-CAPP) Treatment

**FIGURE 1:**
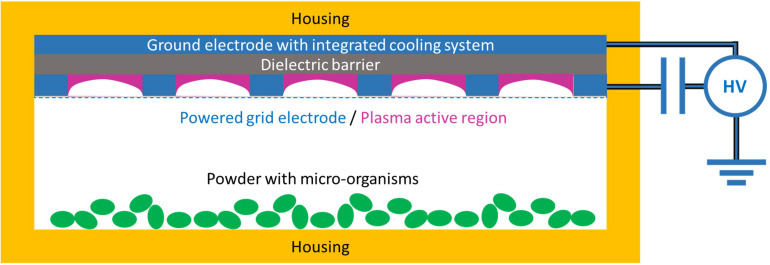
Surface micro discharge (SMD)–cold atmospheric pressure plasma (CAPP) device (schematic drawing).

Home cricket powder was exposed to cold plasma to evaluate antimicrobial effectiveness. An SMD device (Figure 1) fully developed and constructed by the Institute of Systems engineering in collaboration with the Institute of Life Technologies, HES-SO Valais-Wallis was described in detail before (Pina-Pérez et al., 2020). In brief, plasma was ignited in ambient air at atmospheric pressure using an electrical power generator (Titan^TM^ Series, Compact Power Company, United States) at 10 kHz frequency and a high voltage transformer (Swiss Trafo Josef Betschart AG, Switzerland). The high-voltage-powered, planar grid electrode (total surface = 150 cm^2^, grid opening = 9.8 × 9.4 mm) was mounted compactly with a Teflon dielectric barrier and a ground electrode (Figure 1). A quantity of 0.1 g insect powder was spread on sterile glass slides (5 mg powder per cm^2^) and exposed to the plasma at a 6-mm distance to the powered electrode. The discharge power applied was 1.3, 1.8, 2.2, and 3.3 W, corresponding to a plasma power density of 8.7, 12.0, 14.7, and 22.0 mW/cm^2^, respectively. All samples were treated for 5 min. The temperature of the ground electrode was controlled by a water-cooling cycle and was traced with a K-type thermocouple (Thermocoax, France). This temperature determines the temperature in the closed chamber and the sample. Temperatures between 25 and 30°C were detected.

Samples were treated in triplicate for each processing condition. Immediately after CAPP treatments, samples were used for microbiological analysis.

### Thermal Treatment

For thermal treatments, 10 g of *A. domesticus* powder was placed in hermetically closed glass flasks and subjected to a thermal treatment in a FOB2-TS steam sterilizer (Fedegari S.A., Switzerland). The average chamber temperature was developed stepwise and held 30 min at 75°C, 10 min at 110°C, and 20 min at 121°C before vacuum cooling of the chamber to 20°C. The temperature increase in the chamber was reached within 2 min by steam injection at a suited pressure. The samples were not in contact with the steam and were heated indirectly to the equilibrium temperature. Afterward, flasks were removed from the autoclave chamber, and the powder was prepared for microbiological analysis. The thermal treatment was done in triplicate.

### Microbiological Analysis of Samples

Crude powdered samples of *A. domesticus* were prepared in triplicate for microbiological load analysis (suspensions of 1 g/10 ml buffered peptone water, BPW) (Sigma Aldrich, SA, Switzerland). A serial dilution procedure [in sterile BPW 1% (w/v)] of each replicate was carried out, with a final microbial load value estimation as colony-forming units (CFU) per gram (average ± standard deviation), obtained by viable plate count analysis.

Total mesophilic bacterial counts and *Enterobacteriaceae* counts were analyzed in all samples. Sterile Tryptic Soy Agar (TSA) (Sigma Aldrich, SA, Switzerland) was used to estimate total microbial counts from crude powder samples. Specifically, Violet Red Bile Glucose Agar (VRBGA) was used as selective media to estimate viable *Enterobacteriaceae* bacteria in all assays. Microbiological analysis was carried out identically to estimate the number of CFU per gram of powder in untreated, crude powder samples, dry heat-treated samples, and cold plasma processed powders.

Tubes with the powder dispersions were heated in a water bath at 95°C for 15 min and the dispersion was spread on TSA plates. Colonies were transferred to fresh agar plates for isolation (2×). Plates with purified colonies of microorganisms were sent to the Marbitec AG (Riehen, Switzerland) for further analysis. The matrix-assisted laser desorption/ionization-time of flight (MALDI-TOF) mass spectrometry to detect the mass-to-charge ratio (m/z) of molecules was described before (Carbonnelle et al., 2011; Ziegler et al., 2012; Starostin et al., 2015). In brief, material from the colonies transferred to a carrier will be desorbed and partially ionized by a laser. Time-of-flight analysis and mass spectrometry offer possibilities for detecting the unique mass spectral fingerprint of a microbial strain. Fingerprints were compared to biomarkers (super spectrum) reported in the SARAMIS database (AnagosTec GmbH, Potsdam, Germany) and ribosomal proteins (PAPMID^TM^ database). The identification was accepted at a homology of ≥90% with at least one of the databases.

### Lipid-Oxidation Analysis

Considering the radical oxygen species (ROS) formation in the cold plasma “active cloud” in SMD-CAPP treatments, the assessment of possible breakage (hydrolysis) and alteration of PUFA present in *A. domesticus* powder samples is required as a food quality–nutritional preservation indicator. Lipid peroxidation is defined as a chain reaction initiated by the hydrogen abstraction, or ROS addition, resulting in the oxidative damage of PUFA (Repetto et al., 2012). Propagation (peroxyl radicals lead to the production of organic hydroperoxides) implies that once the process is initiated, it can result in the conversion of numerous PUFA to lipid hydroperoxides (ROOH) (first stable products of lipid peroxidation reaction). So, FFA profile modification can be considered as an evidence of autoxidation (free radical reaction) and hydrolytic rancidity.

In the present study, lipid oxidation was quantified using a free-fatty acids detection kit provided by Sigma-Aldrich (Reference: MAK044). Briefly, the concentration of FFA (C8 and longer) is determined by a coupled enzyme assay, which results in a chromophore product (570 nm)/fluorometric (ʎ_ex_ = 535/ʎ_em_ = 587 nm), proportional to the FFA concentration present. Palmitic acid (C16:0) was used as a standard for the quantification of FFA. A standard curve was prepared for analysis with concentrations at 0, 0.2, 0.4, 0.6, 0.8, and 1 nmol.

Samples of *A. domesticus* powder were prepared as follows: First, 10 mg of powder was homogenized in 200 μl of a 1% (w/v) Triton X-100 in chloroform solution. Samples were centrifuged at 13,000 × *g* for 10 min to remove insoluble material. Organic phases were collected and air-dried at 50°C to remove chloroform. Residues of chloroform were removed by vacuum dry for 30 min. Dried lipids were dissolved in 200 μl of fatty acid assay buffer by vortexing extensively for 5 min. Accordingly, the different sample suspensions (crude powder, heat-treated, and CAPP-treated) were used in the analysis, and the concentration of FFA was calculated (Eq. 1):

(1) $$C\,\left(n\,m\,o\,l\,e\right)=\frac{S_{a}}{S_{v}}$$

Where S_a_ is the amount of FFA in an unknown sample (nmol) read from the calibration curve and S_v_ is the sample volume (μl) added to reaction well; C is the concentration of FFA in a sample (nmol/μl).

### Statistical Analysis

All treatments were applied in triplicate. Results were tested for normality and homogeneity before calculating mean values and standard deviations with Statgraphics Centurion XVIII (Statgraphics Inc., United States). An analysis of variance (ANOVA) was carried out to determine the significance of differences (*p* < 0.05) between means, applying Turkey honest significant difference (HSD) test. In figures, the mean variability of data was indicated by the standard deviation.

## Results and Discussion

### Microbial Decontamination

The total microbial count in crude *A. domesticus* powder was 1.9 × 10^6^ cfu/g, and the count of *Enterobacteriaceae* was close to 1.1 × 10^6^ cfu/g. Similar loads were detected in *Hermetia illucens* larvae powder, in detail 1.6 × 10^7^ cfu/g for total aerobic mesophilic bacteria, and 1.2 × 10^6^ cfu/g for *Enterobacteriaceae* (Kashiri et al., 2018); in *Tenebrio molitor* powder, 5.2 × 10^7^ cfu/g for the total microbial count were found (Bußler et al., 2016).

In the present study, the initial microbial load was significantly reduced by thermal (121°C for 20 min) and non-thermal SMD-CAPP treatment (1.3–3.3 W discharge power for 5 min). Mesophilic bacteria were reduced by 2.3 ± 0.4 log_10_ cycles with the heat treatment and 1.4 ± 0.1 log_10_ cycles with the SMD-CAPP treatment. The reduction of *Enterobacteriaceae* in *A. domesticus* powder was significantly higher with the SMD-CAPP treatment (1.9 ± 0.2 log_10_ cycles with a plasma power density of 22.0 mW/cm^2^).

**FIGURE 2:**
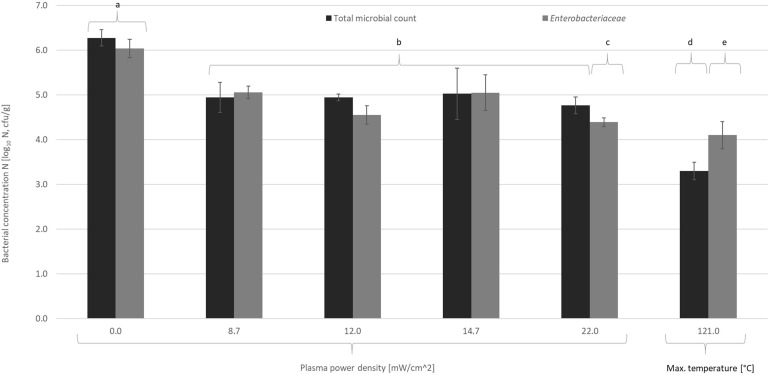
Total bacterial count and *Enterobacteriaceae* in *A. domestica* powder processed 5 min with a surface micro discharge–cold atmospheric pressure plasma (SMD-CAPP) or treated at a maximum temperature of 121°C with dry heat. Significantly different values (*p* < 0.05) were classified, and the classes are indicated with letters.

The increase of the discharge power from 1.3 to 3.3 W or of the discharge power density from 8.7 to 22.0 mW/cm^2^, respectively, does not contribute to higher effectiveness (Figure 2). Plateau values have been reported earlier (Butscher et al., 2016; Beyrer et al., 2020a) and might be caused by protective factors, such as shadowing or encapsulation (Kim et al., 2014; Liao et al., 2017), heterogeneity of age and resistance of the microbe population (Liao et al., 2017), or antioxidant effects (Beyrer et al., 2020a).

Bußler et al. (2016) achieved similar reductions of the total microbial count in *T. molitor* powder by using an SDBD-CAPP (surface dielectric-barrier CAPP, voltage of 8.8 kVpp, frequency of 3.0 kHz), being ≈2 log_10_ reduction in 5 min or 3 log_10_ reductions in 15 min. Rumpold et al. (2014) decontaminated a mealworm powder with a 1,200-W microwave-powered remote plasma and achieved >5 log_10_ reductions of the total bacterial count in 10 min exposure time.

The gas composition [pure helium, argon, nitrogen, or mixtures (e.g., air)], direct vs. indirect plasma exposure, treatment time, and plasma density are essential factors triggering the inactivation effectiveness. For example, Rumpold et al. (2014) identified an indirect, remote cold plasma as more efficient than a direct plasma (>5 log_10_ reductions by remote microwave plasma, 2.45 GHz, 1.2 kW total power, 10 min vs. undetectable inactivation with a plasma jet, 27.12 MHz, 20 W total power).

In *A. domesticus* powder, bacterial spores from *Bacillus cereus* (>99.9% spectra homology), *Bacillus subtilis* (99.9% spectra homology), and *Bacillus megaterium* (96.0% spectra homology) were detected before and after the thermal and SMD-CAPP treatment. Conditions of the thermal or SMD-CAPP treatment are not satisfying for the inactivation of spores of such microorganisms.

An increased plasma density can increase the inactivation effect for *B. subtilis* spores on a flat, smooth surface. In contrast, the presence of particles as in starch or algae powder reduces the inactivation of spores or living cells (Beyrer et al., 2020a; Pina-Pérez et al., 2020). With insect powders, the limitations are comparable, while the protective principle might vary and can involve shadowing or/and reducing the chemical potential of the plasma *via* reactions with other compounds of the matrix than the microorganisms. It was proposed to reduce shadowing effects by fluidizing the powder (Butscher et al., 2016).

Thermal treatments do not solve the problem of contaminations with spores in insect products in general. Grabowski and Klein (2016) found 2–4 log_10_ cycles of *Bacillus* spp. spores in an insect product after 30 min of boiling and drying at 100°C. *B. cereus*, *B. thuringiensis*, and *B. cytotoxicus* were isolated from processed insects (Garofalo et al., 2017) and represent a food safety issue when spores germinate and bacteria produce toxins (Lima et al., 2011; Huang et al., 2020). A tyndallization process for the germination of dormant spores before the inactivation might be an effective strategy to improve the safety of food such as rice (Kim et al., 2012) and could be applied for insect powder containing products.

Considering the energy consumption in thermal and plasma treatments, approximately 2,706 kJ/kg are required for heating the powder to 121°C (Holdsworth and Simpson, 2007), while with 8.7 mW/cm^2^ during 5 min, equal to 500 kJ/kg would be required for the SMD-CAPP treatment. A comprehensive energy balance must consider further factors, such as energy losses at the steam or plasma production and energy for drying subsequently to wet heat treatments.

### Quantification of Free Fatty Acids

*A. domesticus* is a valuable source of unsaturated fatty acids and contains, on average, 23–26% of monounsaturated fatty acids (MUFA), 28–31% of PUFA, and 42–47% of saturated fatty acids (SFA). The FA profile is determined by oleic acid (C18:1, cis 9; 22–24.7%), palmitic acid (C16:0; 26.28–27.46%), and linoleic acid (C18:2, cis 9, 12; 26.74–30.13%) (Kulma et al., 2019). In the present study, FFA concentration variation (calibration with palmitic acid) was traced as an indicator of the hydrolytic potential of an SMD-CAPP. A significant increase (*p* < 0.05) of the FFA concentration was found for the powder treated at the highest plasma density of 22.0 mW/cm^2^ (Table 2). Insignificant hydrolysis was observed for samples processed at 8.7, 12.0, and 14.7 mW/cm^2^ (5 min). So, according to our results, it can be said that SMD-CAPP treatment conditions have demonstrated to be intensive enough to reduce microbial load >1 log_10_ cycles in *A. domesticus* powder (8.7–14.7 mW/cm^2^) and do not increase the concentration of primary products of lipid oxidation.

**TABLE 2:** Free fatty acid (FFA) concentration of *A. domesticus* powders before (control) and after surface micro discharge–cold atmospheric pressure plasma (SMD-CAPP) treatments.

**Plasma density (mW/cm^2^)**	**FFA concentration (% w/w DM)**
0 (control)	12.9 ± 0.7^a^
8.7	13.7 ± 0.7^a^
12.0	13.3 ± 0.7^a^
14.7	13.2 ± 0.7^a^
22.0	16.3 ± 0.8^b^

*^*a,b*^Different superscript letters correspond to significantly different values.*

Lipid oxidation and increased rancidity have been previously reported, depending on the matrix and plasma conditions applied (Gavahian et al., 2018; Lee et al., 2018; Pérez-Andrés et al., 2020). According to Gebremical et al. (2019), an increase of FFA in peanuts was observed with a power of 36 W in a DBD plasma. Also, Yepez and Keener (2016) reported a significant increment of different FFA (C18:1, n-9; C18:0; C16:0) after an extended exposure of 1–12 h of soybean oil to a plasma powered with 90 kV. Albertos et al. (2017) detected an increase of FFA concentrations (C16:0; C18:1, n-9; C20:5, n-3; C22:6n-3) in mackerel after a DBD CAPP treatment with a discharge voltage of 70 and 80 kV for 1, 3, and 5 min. So, it is supposed that both the plasma power and treatment time influence the FFA content in TG-rich food.

Surowsky et al. (2016), summarized that regarding most sensitive poly-unsaturated FA in food, Linoleic (C18:2) and α-Linolenic (C18:3) were modified in a 20-min CAPP and the activation energy of the double-bond hydrogenation was 422 kJ/mol. In wheat flour, the linoleic and linolenic acid concentration was reduced significantly by treatment of 2 min already, applying a power of 40–90 W for igniting the plasma in the air (Bahrami et al., 2016). In conclusion, a low dosage of the plasma, such as applied in the current study, characterized by a low discharge power or plasma density, is conditional for preventing hydrolysis of TGs and increasing the FFA content in food.

## Conclusion

The treatment at a very low plasma density of only 8.7–22.0 mW/cm^2^ with an SMD setup results in a 1–2 log_10_ reduction of the total microbial count in an *A. domesticus* powder (mainly in the reduction of *Enterobacteriaceae*), and this at almost no increase of the FFA content. Shadowing might inhibit the inactivation of near-surface bacteria, and fluidization might improve the efficiency of the treatments. The increase of the plasma power does not increase the inactivation. An intensification of the plasma treatment results in an increased risk of the hydrolysis of triglycerides or the oxidation of mono- or poly-unsaturated fatty acids. Further advantage can be provided through energy savings comparing plasma with thermal treatments.

## Data Availability Statement

The raw data supporting the conclusions of this article will be made available by the authors, without undue reservation, to any qualified researcher.

## Author Contributions

MP-P contributed to this work with project acquisition, methodology, investigation, formal analysis, and writing the manuscript. DR contributed with consulting during the investigation and critically reviewing the manuscript. CE contributed to engineering the plasma equipment and plasma diagnostics. MB was responsible for the funding acquisition, concepts, data analysis, editing the manuscript, supervision, and project administration. All authors contributed to the article and approved the submitted version.

## Conflict of Interest

The authors declare that the research was conducted in the absence of any commercial or financial relationships that could be construed as a potential conflict of interest.
